# Effect of Seyoeum on Obesity, Insulin Resistance, and Nonalcoholic Fatty Liver Disease of High-Fat Diet-Fed C57BL/6 Mice

**DOI:** 10.1155/2017/4658543

**Published:** 2017-09-11

**Authors:** Hyun-Young Na, Mi Hyeon Seol, Mia Kim, Byung-Cheol Lee

**Affiliations:** ^1^Department of Clinical Korean Medicine, Graduate School, Kyung Hee University, 26 Kyungheedae-ro, Dongdaemun-gu, Seoul 02447, Republic of Korea; ^2^Department of Cardiovascular and Neurologic Disease (Stroke Center), College of Korean Medicine, Kyung Hee University, 23 Kyungheedae-ro, Dongdaemun-gu, Seoul 02447, Republic of Korea

## Abstract

**Background:**

This study was performed to evaluate the effect of Seyoeum (SYE), a novel herbal meal replacement, on insulin resistance and nonalcoholic fatty liver disease (NAFLD) in obese mice fed with a high-fat diet (HFD).

**Methods:**

SYE contained six kinds of herbal powder such as* Coix lacryma-jobi*,* Oryza sativa*,* Sesamum indicum*,* Glycine max*,* Liriope platyphylla,* and* Dioscorea batatas*. Male C57BL/6 mice were divided into four groups: normal chow (NC), HFD, SYE, and HFD plus SYE (HFD + SYE). The mice in groups other than NC were fed HFD for 9 weeks to induce obesity and then were fed each diet for 6 weeks. Clinical markers related to obesity, diabetes, and NAFLD were examined and gene expressions related to inflammation and insulin receptor were determined.

**Results:**

Compared with HFD group, body weight, serum glucose, serum insulin, HOMA-IR, total cholesterol, triglyceride, epididymal fat pad weight, liver weight, and inflammatory gene expression were significantly reduced in SYE group. Insulin receptor gene expression increased in SYE group.

**Conclusions:**

Based on these results, we conclude that SYE improved obesity and insulin resistance in high-fat fed obese mice. Our findings suggest that SYE could be a beneficial meal replacement through these antiobesity and anti-insulin resistance effects.

## 1. Introduction

Obesity is a major risk factor for many chronic diseases including insulin resistance, type 2 diabetes, atherosclerosis, and nonalcoholic fatty liver disease (NAFLD) [1]. Insulin resistance is a common pathogenic event that links obesity with metabolic syndrome and NAFLD [2]. High insulin concentration in insulin resistance stimulates lipogenesis through the activation of sterol regulatory element-binding protein 1 (SREBP-1c) [3], which inhibits insulin receptor substrate 2 (IRS-2) mediated insulin signaling [4]. NAFLD activates gluconeogenesis leading to hepatic insulin resistance [5]. The excess accumulation of lipid in adipose tissue and liver accompanies a chronic, subacute state of inflammation, which can be seen in the involved tissues and systemically, in terms of elevated circulating levels of inflammatory markers [1].

A number of strategies including exercise, a structured dietary plan, cognitive behavioral therapy, pharmacotherapy, and bariatric surgery are available [6]. Yet, it is hard to make a significant and sustainable difference by lifestyle interventions, and pharmacologic or surgical interventions should be considered only in certain patients [7].

Meal replacement is commonly used as adjunctive therapy in the diet of various treatments for obesity. Meal replacement is convenient and has a low risk of side effect, providing a fixed amount of calorie, dose, and nutritional content [8]. Some studies suggest it for an effective option for long-term compliance or improvement in metabolic risk factors of outpatients [9–13]. Clinical studies showed that partial meal replacement therapy had better weight loss and long-term effects compared to conventional calorie restriction [14]; however, most of the studies about meal replacement have targeted weight loss effects for humans. Besides, there are few studies about effects of using meal replacements only, and there are very few mechanism studies about effects of meal replacements on metabolic risk factors. So in this study, we investigated the effect of Seyoeum (SYE), a new meal replacement which consisted of six kinds of herbs, on obesity and insulin resistance, on high-fat diet (HFD) fed C57BL/6 mice.

## 2. Materials and Methods

### 2.1. Preparation of Seyoeum

The herbs were obtained from the Department of Pharmaceutical Preparation of Korean Medicine, Korean Medical Hospital, Kyung Hee University, Seoul, Korea. SYE is a combination of herbal powder (i.e.,* Coix lacryma-jobi* Linné var.* ma-yuen* Stapf,* Oryza sativa* Linné,* Sesamum indicum* Linné,* Glycine max* Merrill,* Liriope platyphylla* F. T. Wang & Tang, and* Dioscorea batatas* Decne.) at a ratio of 1 : 1 : 2 : 2: 2 : 1.5. Five kinds of herbs except* Liriope platyphylla* were roasted to develop flavors. They were dried and grounded to form a powder. The powder was kneaded in water, dried in feed form, and fed. Some SYE powder was kneaded with the same weight of HFD and dried in feed form.

### 2.2. Measurements of Calorie and Nutritional Component of Seyoeum

SYE was requested for analysis of nutritional components by the Korea Advanced Food Research Institute. Twelve components including calorie, carbohydrate, crude protein, crude fat, sodium, sugars, saturated fat, trans fat, cholesterol, dietary fiber, iron, and calcium were analyzed.

### 2.3. Animal Model and Treatment

Six-week-old male C57BL/6J mice were purchased from the Central Lab. Animal Inc. (Seoul, Korea). They were in a moisture controlled room (40–70%) with a 12-hour light-dark cycle and allowed access to water and diet ad libitum. After 1-week period of acclimation, every mouse except normal chow (NC) diet group were fed a HFD (60% energy by fat) known for causing obesity for a 9-week period. The mice were divided into four groups according to diet: NC group (*n* = 6), HFD group (*n* = 6), SYE group (*n* = 5), and HFD plus SYE group (HFD + SYE, *n* = 5). Then the mice were fed one of the HFD, SYE, or HFD plus SYE for 6 weeks. HFD plus SYE were mixed at 1 : 1 ratio. The body weight of each mouse was measured at the beginning and before the final sampling. The total amount of food consumption was recorded every day. To assess the food intake, the total consumption of food during a day was measured in every cage. Then the 1-day consumption of each mouse was calculated by dividing into the number of the mice in each cage. At week 16, the mice were sacrificed and the weights of livers and the epididymal fat pads were measured. This study was approved by the Institutional Animal Care and Use Committee of Kyung Hee University, Seoul.

### 2.4. Oral Glucose Tolerance Test (OGTT)

At week 14, mice were fasted for 14 hours and glucose (2 g/kg body weight) dissolved in water was administered to all the mice orally. Blood samples were taken from tail vein at 0, 30, 60, and 120 minutes after glucose administration. Glucose was measured using a strip-operated blood glucose sensor (ACCU-CHEK Performa. Castle Hill, NSW, Australia). The area under the curve (AUC) of glucose in the OGTT was calculated from measurements taken before (0 minutes) and after (up to 120 minutes) glucose administration on the basis of the trapezoidal rule, which is a method used to approximate a definite integral by evaluating the integrand at two points.

### 2.5. Biochemical Assays

At week 14, blood was collected from the tail vein of each mouse. Plasma insulin concentration was quantified using an ultrasensitive mouse insulin ELISA kit (Crystal Chem INC., Chicago, IL, USA). Glucose and insulin levels were measured and insulin resistance was assessed by a homeostatic model assessment of insulin resistance (HOMA-IR). HOMA-IR was calculated using the following formula: HOMA-IR = Fasting blood glucose (mg/dl) × Fasting insulin (ng/ml) × 0.0717225161669606. At week 16, blood was collected from the hearts, while the mice were under anesthesia with diethyl ether. Aspartate transaminase (AST), alanine transaminase (ALT), total cholesterol, high density lipoprotein (HDL) cholesterol, and triglyceride levels were measured.

### 2.6. Morphological Analysis of Liver

The liver samples were immersion-fixed in 10% buffered formalin and embedded in paraffin for study by light microscopy. Two sections per animal, 5 *μ*m thick (at an interval of 100 *μ*m), were stained with periodic acid-Schiff (PAS) reagent. The sections were photographed under a photomicroscope (Olympus BX-50; Olympus Optical, Tokyo, Japan).

### 2.7. RNA Isolation and Quantitative Real-Time Polymerase Chain Reaction Analysis

At week 16, the mice were sacrificed and the epididymal fat pads were dissected. RNA extraction was performed using a Mini RNA Isolation IITM (Zymo Research, Orange, CA, USA). RNA from liver tissue was extracted using Trizol reagent. To evaluate the gene expression levels, we performed quantitative real-time polymerase chain reaction (qRT-PCR). The complementary DNA (cDNA) was synthesized using an Advantage RT for PCR Kit (Clontech, Palo Alto, CA, USA). The sequences of primes used in this study are shown in Table 1. PCR was carried out in a 7900HT Fast Real-Time PCR System (Applied Biosystems®, Foster City, CA, USA). For gene expression analysis, threshold cycle (Ct) of each gene was calculated by SDS Software 2.4 (Applied Biosystems, Foster City, CA, USA); then relative quantitation (RQ) was performed relative to glyceraldehyde-3-phosphate dehydrogenase (GAPDH, housekeeping gene). The fold change was calculated according to NC group which was considered as 1.

### 2.8. Statistical Analysis

Statistical analyses were performed using GraphPad PRISM 6 (Graphpad software inc., San Diego, CA, USA). Statistical comparisons between the groups were performed with one-way analysis of variance (ANOVA), followed by Tukey's post hoc test. The data are presented as mean ± SE. A two-tailed *P* value of <0.05 was considered statistically significant.

## 3. Results

### 3.1. Calorie and Composition of SYE

The calorie value of SYE was 398.3 kcal/100 g. The amounts of carbohydrate, crude protein, and crude fat per 100 g of SYE were 61.7 g, 19.3 g, and 12.7 g, respectively (Table 2).

### 3.2. SYE Reduced Body Weight, Epididymal Fat Weight, and Liver Weight in HFD Mice

**Figure 1:**
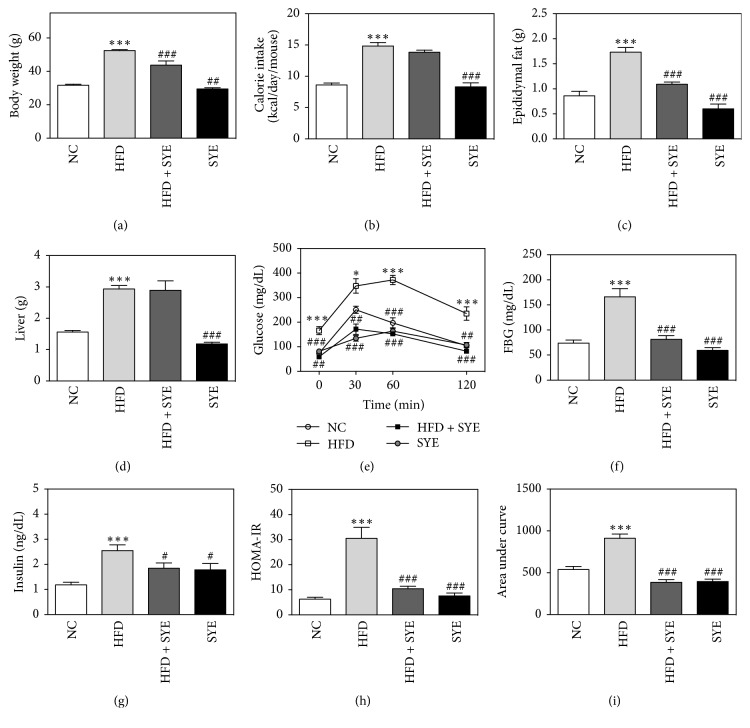
The changes by Seyoeum on body weight, calorie intake, epididymal fat, liver weight, calorie intake, OGTT, fasting blood glucose, insulin, HOMA-IR, and AUC. *N* = 6 in NC and HFD groups, and *N* = 5 in SYE and HFD + SYE groups. ^*∗∗∗*^*P* < 0.001 versus NC group; ^#^*P* < 0.05 and ^###^*P* < 0.001 versus HFD group. ## means *P* < 0.01 versus HFD group. *∗* means *P* < 0.05 versus NC group.

The body weight of HFD mice significantly increased to 52.48 ± 0.56 g, while that of NC mice was 31.71 ± 0.90 (*P* < 0.001). SYE significantly lowered body weight compared to HFD group, as much as 43.8% in SYE group (29.48 ± 4.95, *P* < 0.001) and 16.7% in HFD + SYE group (43.72 ± 7.59, *P* < 0.01) (Figure 1(a)). The amount of food intake (data not shown) and calorie intake were also examined to determine if the weight loss was due to a decrease in the intake. Compared with the HFD group, there were significant decreases both in food intake and calorie intake in the SYE group (*P* < 0.001) but not in the HFD + SYE group (Figure 1(b)). Weight of epididymal fat pad significantly increased by HFD compared to NC group (*P* < 0.001) and SYE significantly reduced the epididymal fat as much as 65.9% in SYE group (*P* < 0.001) and 37.0% in HFD + SYE group (*P* < 0.001) (Figure 1(c)). Weight of liver significantly increased in HFD group compared to that in NC group (*P* < 0.001). In SYE group, weight of liver was significantly lower than HFD group (*P* < 0.001), but in HFD + SYE group the liver weight was lower without significance (Figure 1(d)).

### 3.3. SYE Improved Hyperglycemia and Insulin Resistance in HFD Mice

In HFD group, blood glucose levels were elevated compared to NC group at 0, 30, 60, and 120 minutes (*P* < 0.05 at 30 min and *P* < 0.001 at others). SYE significantly reduced fasting blood glucose levels as much as 64.3% in SYE group (*P* < 0.001) and 51.0% in HFD + SYE group (*P* < 0.01), compared to HFD group. At the other time points, SYE made significant reduce in both groups (Figures 1(e) and 1(f)). Fasting insulin level, HOMA-IR, and AUC were significantly increased in HFD group compared to NC group (*P* < 0.001) and were reduced significantly in SYE group and HFD + SYE group compared to HFD group (Figures 1(g), 1(h), and 1(i)).

### 3.4. Effects of SYE on Liver Enzyme, Lipid Levels in Serum, and Lipid Accumulation in Liver

**Figure 2:**
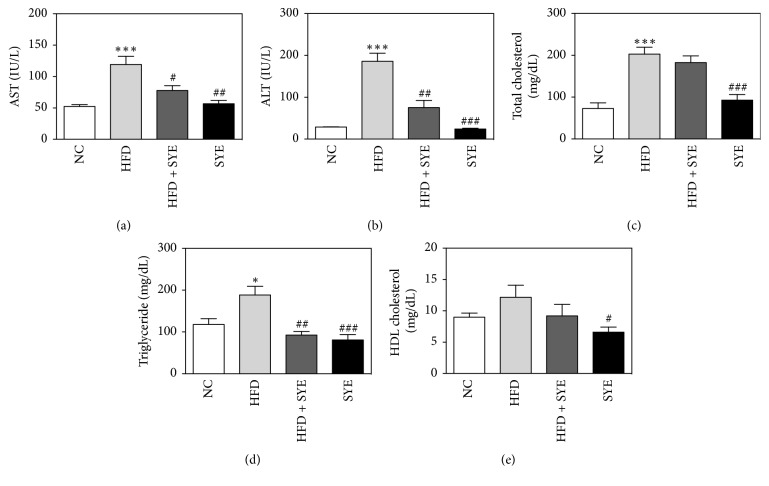
The changes of liver enzyme and lipid levels by Seyoeum. Blood samples were obtained at week 16. *N* = 6 in NC and HFD groups, and *N* = 5 in SYE and HFD + SYE groups. ^*∗*^*P* < 0.05 and ^*∗∗∗*^*P* < 0.001 versus NC group; ^#^*P* < 0.05, ^##^*P* < 0.01, and ^###^*P* < 0.001 versus HFD group.

**Figure 3:**
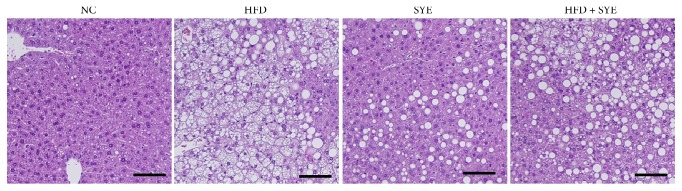
Effects of Seyoeum on liver in mice. Liver sections from representative mice of each group (periodic acid-Schiff, scale bar = 100 *μ*m).

In HFD group, both AST and ALT significantly increased (*P* < 0.001) compared to NC group but, however, significantly decreased in SYE group (*P* < 0.01 for AST and *P* < 0.001 for ALT) compared to HFD group (Figures 2(a) and 2(b)). HFD significantly raised total cholesterol and triglyceride (*P* < 0.001 for total cholesterol and *P* < 0.05 for triglyceride) and decreased HDL cholesterol level without significance, compared to NC. SYE significantly lowered total cholesterol levels (*P* < 0.001) and lowered triglyceride level, compared to HFD. HFD + SYE mice decreased triglyceride level (*P* < 0.01), but did not show any differences among total cholesterol, HDL cholesterol (Figures 2(c), 2(d), and 2(e)). Since the liver TG level showed a tendency to be lowered by SYE treatment, histological analysis was performed by staining the liver with periodic acid-Schiff. Marked lipid accumulation was observed in hepatocytes in the HFD group, whereas there was little lipid accumulation in hepatocytes in the HFD + SYE and SYE groups (Figure 3).

### 3.5. SYE Suppressed the Inflammatory Gene Expression in Epididymal Adipose Tissue of HFD Mice

**Figure 4:**
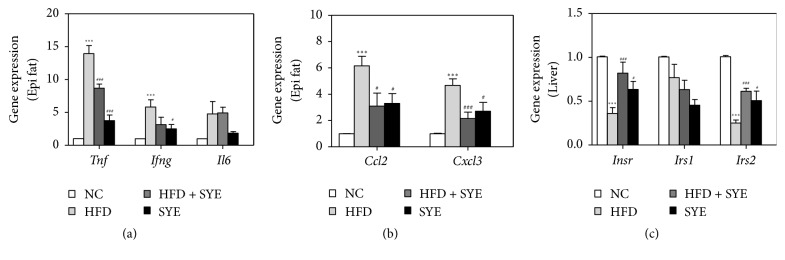
The changes by Seyoeum on inflammatory cytokine and chemokine gene expression in adipose tissue, and insulin receptor gene expression in liver tissue. Adipose and liver tissue was obtained at week 16 and quantitative RT-PCR was used for measuring gene expression. Gene expression was normalized to that of GAPDH. *N* = 6 in NC and HFD groups, and *N* = 5 in SYE and HFD + SYE groups. ^*∗*^*P* < 0.05 and ^*∗∗∗*^*P* < 0.001 versus NC group; ^#^*P* < 0.05 and ^###^*P* < 0.001 versus HFD group.

Because adipose tissue releases inflammatory cytokines which induce insulin resistance, we analyzed the inflammatory cytokine gene expression in epididymal fat. The genes examined were tumor necrosis factor alpha (TNF-*α*), interferon gamma (IFN-*γ*), and interleukin-6 (IL-6) for inflammatory cytokines and monocyte chemotactic protein-1 (MCP-1) and Chemokine (C-X-C motif) ligand 3 (Cxcl3) for chemokines. TNF-*α* and IFN-*γ* expressions in the HFD group significantly increased compared to NC group (*P* < 0.001). SYE significantly decreased TNF-*α* expression in both SYE and HFD + SYE groups (*P* < 0.001) and significantly lowered IFN-*γ* expression in SYE group only (*P* < 0.05). There were no significant differences in IL-6 expression between HFD and NC group, but also between HFD and SYE or HFD + SYE group (Figure 4(a)). HFD significantly increased both MCP-1 and Cxcl3 expressions compared to NC group (*P* < 0.001), and SYE significantly decreased the levels in both SYE (*P* < 0.05) and HFD + SYE groups (*P* < 0.05 for MCP-1 and *P* < 0.001 for Cxcl3) (Figure 4(b)).

### 3.6. SYE Improved the Insulin Receptor Gene Expression in Liver Tissue of HFD Mice

Obesity changes the secretion of adipose tissue inflammatory cytokines, which modulate insulin signaling, so we analyzed the insulin signaling gene expression including insulin receptor (IR), insulin receptor substrate 1 (IRS-1), and IRS-2 in liver tissue. The IR and IRS-2 expression levels were significantly decreased in HFD group relative to the NC group (*P* < 0.001), and SYE significantly increase the levels compared to HFD group in both SYE (*P* < 0.05) and HFD + SYE groups (*P* < 0.001). In case of IRS-1 expression level, there were no significant differences among the groups (Figure 4(c)).

## 4. Discussion

In an obese state, excessive accumulation of lipids in adipose tissue activates inflammatory cytokines and chemokines secretion, which causes chronic low-level inflammation and insulin resistance. Therefore, obesity becomes a risk factor for many diseases, including metabolic syndrome, cardiovascular disease, and cancer [1, 15, 16]. If we can block the systemic inflammation and insulin resistance as well as the reduction of fat cells, it can be expected to lower the risk of complications due to obesity. In this study, we tried to evaluate the effects of a new meal replacement on obesity and metabolic syndrome in mice, using clinical markers including body weight, glucose level, insulin level, HOMA-IR, lipid level, epididymal fat pad weight, liver weight, and gene expressions related to inflammation and insulin receptor.

“Sunsik” is a substitute food in Korea that takes a variety of roasted grains, vegetables, nuts, and other foods into water or milk [17]. The authors developed a meal replacement based on the idea of Sunsik. We selected candidate herbs by screening for common kinds of whole grains used in Sunsik and herbs that have been clinically and experimentally proven to be effective against obesity, diabetes, or NAFLD. Then, some formulations with good flavor were developed to increase compliance, and blind tasting was given to 10 testers to confirm the highest preference.

For the six selected herbs, either themselves or the ingredients were reported to be effective in obesity, IR, dyslipidemia, or NAFLD.* Coix lacryma-jobi* has effects on obesity and hyperlipidemia through modulating TNF-alpha and leptin [18], and* Oryza sativa* decreases hepatic fat accumulation, hyperlipidemia [19], and hepatic steatosis [20]. Liriope platyphylla improves fat accumulation and glucose regulation [21] and stimulates insulin secretion and suppresses fatty liver formation [22].* Dioscorea batatas* has beneficial activities against obesity through decreasing expression of inflammatory cytokines [23] and has an effect on insulin resistance [24]. Sesamol, a component of* Sesamum indicum*, has beneficial activities against hypercholesterolemia, insulin resistance, and hepatic steatosis [25]. The common name for Glycine max is soy, and soy beta- conglycinin improved metabolic abnormalities in a NAFLD rat [26] and improved hepatic insulin resistance [27]. SYE significantly decreased body weight, liver weight, and epididymal fat weight of HFD fed mice. HFD + SYE diet also reduced body weight and epididymal fat weight without significant calorie difference from HFD, so the weight loss effect might be resulted from not only reduced calorie intake but also pharmacological effect of SYE itself. High-fat diet induces weight gain of liver, through accumulation of cholesterol and triglyceride in the liver [28]. Liver fat is highly and linearly correlated with all components of the metabolic syndrome, independent of obesity [29]. Epididymal fat is equivalent to human visceral fat, and visceral fat secretes adipokines and cytokines related to fat metabolism and insulin sensitivity [15]. Visceral fat is known to be more related to metabolic syndrome than body mass index (BMI) [1].

In other similar experiments, lipid decrease was mainly observed [30, 31], but SYE showed significant changes in glucose metabolism. SYE remarkably improved all the indicators related to insulin resistance, including glucose level, serum insulin level, HOMA-IR, and AUC in both SYE and HFD + SYE groups. Through this, we have identified the potential effects of SYE on fat loss and metabolic syndrome. SYE mostly consists of whole grain and has large amounts of dietary fiber and phenolic compounds compared with refined cereal, which could be considered to protect against obesity complications such as cardiovascular disease, cancer, and diabetes [32].

The effect of SYE on the inflammatory gene expression in adipose tissue was prominent in TNF-*α*. TNF-*α* is one of the major local regulators in adipose tissue that contributes to insulin resistance and inflammation [33]. MCP-1 and Cxcl3 are chemokines that promote the adhesion of monocytes and induce low-inflammation, and SYE decreased the expression of MCP-1 and Cxcl3. Cxcl3, also known as the oncogene, interacts with the cell surface chemokine receptor MCP-1, to regulate the migration and adhesion of monocytes and regulate the effect on target cells [34, 35]. By inhibiting TNF-*α*, MCP-1, and Cxcl3 expression in adipocytes, SYE seems to inhibit local inflammatory responses.

IL-6 is a classical inflammatory cytokine considered to be a risk factor for the onset of diabetes [36]. Both TNF-*α* and IL-6 are involved in classical receptor mediated processes, including c-Jun aminoterminal kinase (JNK) and I*κ*B kinase-*β* (IKK-*β*)/nuclear factor-*κ*B (NF-*κ*B), which upregulate inflammatory mediators [37]. SYE significantly inhibited the expression of TNF-*α* but not IL-6. TNF-*α* is mainly produced by activated macrophages [38], whereas IL-6 can be produced in a number of immune cells including activated macrophages and lymphocytes [39]. This suggests that SYE is more effective in inhibiting macrophages than other immune cells. IFN-*γ* activates immune cells, including macrophages, secreted only by T cells and NK cells. The hypothesis that SYE specifically inhibits the action of macrophages could also explain the low effect of SYE on IFN-*γ* in the HFD + SYE group [34, 35]. From these results, we supposed that SYE inhibits immune cell adhesion and reduces insulin resistance and inflammation; activated macrophage especially was likely to be suppressed.

Inhibition of the insulin receptor signaling pathway by inflammation or stress is a key mechanism of insulin resistance. Defects in insulin receptor signaling pathway are observed in most of the systemic insulin resistance [40]. To evaluate insulin signaling, gene expression of IR, IRS-1, and IRS-2 in the liver was analyzed. IR and IRS-2 expressions were increased in the SYE and HFD + SYE groups compared to HFD group. The expression of IRS-1 in the liver appears to be relatively unaffected by obesity. The anti-inflammatory effect of SYE appears to improve the insulin signaling pathway in the liver.

## 5. Conclusions

Based on these results, we conclude that SYE improved obesity, insulin resistance, and NAFLD in high-fat diet-fed obese mice. Our findings suggest that these antiobesity, anti-insulin resistance effects of SYE could be mediated by the suppression of adipose tissue inflammatory cytokines and enhancing the insulin signaling pathway. Further studies related to other mechanisms on inflammatory response in adipose tissue, obesity, and insulin resistance should be taken for the clinical application of SYE.

## Figures and Tables

**Table 1:** Primer sequences.

Target	Primer	Sequence (5′-3′)
TNF-*α*	F	TTCTGTCTACTGAACTTCGGGGTGATCGGTCC
	R	GTATGAGATAGCAAATCGGCTGACGGTGTGGG
IFN-*γ*	F	ACTGGCAAAAGGATGGTGAC
	R	TGAGCTCATTGAATGCTTGG
IL-6	F	AACGATGATGCACTTGCAGA
	R	GAGCATTGGAAATTGGGGTA
MCP-1	F	CCCACTCACCTGCTGCTACT
	R	TCTGGACCCATTCCTTCTTG
Cxcl3	F	AGGCTACAGGGGCTGTTGT
	R	GGGTTGAGGCAAACTTCTTG
IR	F	GAGATGGTCCACCTGAAGGA
	R	GGACAGACATCCCCACATTC
IRS-1	F	AAGCACCTGGTGGCTCTCTA
	R	TCAGGATAACCTGCCAGACC
IRS-2	F	ATACCGCCTATGCCTGTCTG
	R	TGGTCTCATGGATGTTCTGC
GAPDH	F	AGTCCATGCCATCACTGCCACC
	R	CCAGTGAGCTTCCCGTTCAGC

**Table 2:** Calorie and composition of Seyoeum.

Nutrition facts (per 100 g)	Amount
Calorie (kcal)	398.3
Carbohydrate (g)	61.7
Crude protein (g)	19.3
Crude fat (g)	12.7
Sodium (mg)	17.34
Sugars (g)	4.6
Saturated fat (g)	2.2
Trans fat (g)	0.39
Cholesterol (mg)	1.55
Dietary fiber (g)	20.0
Iron (mg)	10.72
Calcium (mg)	480.97

## References

[1] Shoelson S. E., Herrero L., Naaz A. (2007). Obesity, inflammation, and insulin resistance. *Gastroenterology*.

[2] Marchesini G., Marzocchi R., Agostini F., Bugianesi E. (2005). Nonalcoholic fatty liver disease and the metabolic syndrome. *Current Opinion in Lipidology*.

[3] Shimomura I., Bashmakov Y., Ikemoto S., Horton J. D., Brown M. S., Goldstein J. L. (1999). Insulin selectively increases SREBP-1C mRNA in the livers of rats with streptozotocin-induced diabetes. *Proceedings of the National Academy of Sciences of the United States of America*.

[4] Ide T., Shimano H., Yahagi N. (2004). SREBPs suppress IRS-2-mediated insulin signalling in the liver. *Nature Cell Biology*.

[5] Samuel V. T., Liu Z.-X., Qu X. (2004). Mechanism of hepatic insulin resistance in non-alcoholic fatty liver disease. *The Journal of Biological Chemistry*.

[6] Reichert R. G., Reimer R. A., Kacinik V., Pal S., Gahler R. J., Wood S. (2013). Meal replacements and fibre supplement as a strategy for weight loss. Proprietary PGX® meal replacement and PGX® fibre supplement in addition to a calorie-restricted diet to achieve weight loss in a clinical setting. *Biotechnology and Genetic Engineering Reviews*.

[7] Nuffer W. A., Trujillo J. M. (2015). Liraglutide: A New Option for the Treatment of Obesity. *Pharmacotherapy*.

[8] Berkowitz R. I., Wadden T. A., Gehrman C. A. (2011). Meal replacements in the treatment of adolescent obesity: A randomized controlled trial. *Obesity*.

[9] Ashley J. M., St. Jeor S. T., Schrage J. P. (2001). Weight control in the physician's office. *Archives of Internal Medicine*.

[10] Flechtner-Mors M., Ditschuneit H. H., Johnson T. D., Suchard M. A., Adler G. (2000). Metabolic and weight loss effects of long-term dietary intervention in obese patients: Four-year results. *Obesity Research*.

[11] Quinn Rothacker D. (2000). Five-year self-management of weight using meal replacements: Comparison with matched controls in rural Wisconsin. *Nutrition*.

[12] Ditschuneit H. H., Flechtner-Mors M., Johnson T. D., Adler G. (1999). Metabolic and weight-loss effects of a long-term dietary intervention in obese patients. *American Journal of Clinical Nutrition*.

[13] Heber D., Ashley J. M., Wang H.-J., Elashoff R. M. (1994). Clinical evaluation of a minimal intervention meal replacement regimen for weight reduction. *Journal of the American College of Nutrition*.

[14] Heymsfield S. B., Van Mierlo C. A. J., Van Der Knaap H. C. M., Heo M., Frier H. I. (2003). Weight management using a meal replacement strategy: Meta and pooling analysis from six studies. *International Journal of Obesity*.

[15] Scherer P. E. (2006). Adipose tissue: from lipid storage compartment to endocrine organ. *Diabetes*.

[16] Fried S. K., Bunkin D. A., Greenberg A. S. (1998). Omental and subcutaneous adipose tissues of obese subjects release interleukin-6: depot difference and regulation by glucocorticoid. *Journal of Clinical Endocrinology and Metabolism*.

[17] Chung S.-S., Han Y.-S. Consumer's recognition, nutrient composition, and safety evaluation of commercial Sunsik and Saengsik. *Journal of The Korean Society of Dietary Culture*.

[18] Kim S. O., Yun S.-J., Jung B. (2004). Hypolipidemic effects of crude extract of adlay seed (Coix lachrymajobi var. mayuen) in obesity rat fed high fat diet:Relations of TNF-*α* and leptin mRNA expressions and serum lipid levels. *Life Sciences*.

[19] Choi W. H., Um M. Y., Ahn J., Jung C. H., Ha T. Y. (2014). Cooked rice inhibits hepatic fat accumulation by regulating lipid metabolism-related gene expression in mice fed a high-fat diet. *Journal of Medicinal Food*.

[20] Jang H.-H., Park M.-Y., Kim H.-W. (2012). Black rice (Oryza sativa L.) extract attenuates hepatic steatosis in C57BL/6 J mice fed a high-fat diet via fatty acid oxidation. *Nutrition and Metabolism*.

[21] Kim J., Hwang I., Choi S. (2012). Aqueous extract of. *Laboratory Animal Research*.

[22] Lee H. R., Kim J. E., Goo J. S. (2012). Red Liriope platyphylla contains a large amount of polyphenolic compounds which stimulate insulin secretion and suppress fatty liver formation through the regulation of fatty acid oxidation in OLETF rats. *International Journal of Molecular Medicine*.

[23] Gil H.-W., Lee E.-Y., Lee J.-H. (2015). Dioscorea batatas extract attenuates high-fat diet-induced obesity in mice by decreasing expression of inflammatory cytokines. *Medical Science Monitor*.

[24] Kim S., Jwa H., Yanagawa Y., Park T. (2012). Extract from Dioscorea batatas ameliorates insulin resistance in mice fed a high-fat diet. *Journal of Medicinal Food*.

[25] Sharma A. K., Bharti S., Bhatia J. (2012). Sesamol alleviates diet-induced cardiometabolic syndrome in rats via up-regulating PPAR*γ*, PPAR*α* and e-NOS. *Journal of Nutritional Biochemistry*.

[26] Wanezaki S., Tachibana N., Nagata M. (2015). Soy *β*-conglycinin improves obesity-induced metabolic abnormalities in a rat model of nonalcoholic fatty liver disease. *Obesity Research and Clinical Practice*.

[27] Tachibana N., Yamashita Y., Nagata M. (2014). Soy *β*-conglycinin improves glucose uptake in skeletal muscle and ameliorates hepatic insulin resistance in Goto-Kakizaki rats. *Nutrition Research*.

[28] Jayasooriya A. P., Sakono M., Yukizaki C., Kawano M., Yamamoto K., Fukuda N. (2000). Effects of *Momordica charantia* powder on serum glucose levels and various lipid parameters in rats fed with cholesterol-free and cholesterol-enriched diets. *Journal of Ethnopharmacology*.

[29] Kotronen A., Yki-Järvinen H. (2008). Fatty liver: a novel component of the metabolic syndrome. *Arteriosclerosis, Thrombosis, and Vascular Biology*.

[30] Kim S.-S., Seong K.-S., Lee O.-H. (2014). Effects of phytoplant diets on body weight, feces production, body fat, and serum lipid levels in high-fat diet-induced hyperlipidemic rats. *Korean Journal of Food Science and Technology*.

[31] Shin I., Choi H., Ku S., Kim M. (2012). The Effect of Natural Mixture Supplementation on Histopathological and Histomorphometrical aspects in High Fat Diet-induced Obese Mice. *The Korea Journal of Herbology*.

[32] Kim J. Y., Shin J. H., Lee S. S. (2012). Cardioprotective effects of diet with different grains on lipid profiles and antioxidative system in obesity-induced rats. *International Journal for Vitamin and Nutrition Research*.

[33] Trayhurn P., Wood I. S. (2004). Adipokines: inflammation and the pleiotropic role of white adipose tissue. *British Journal of Nutrition*.

[34] Smith D. F., Galkina E., Ley K., Huo Y. (2005). GRO family chemokines are specialized for monocyte arrest from flow. *American Journal of Physiology - Heart and Circulatory Physiology*.

[35] Ahtga S. K., Murphy P. M. (1996). The CXC chemokines growth-regulated oncogene (GRO) *α*, GRO*β*, GRO*γ*, neutrophil-activating peptide-2, and epithelial cell-derived neutrophil-activating peptide-78 are potent agonists for the type B, but Not the type A, human interleukin-8 receptor. *Journal of Biological Chemistry*.

[36] Lee B.-C., Lee J. (2014). Cellular and molecular players in adipose tissue inflammation in the development of obesity-induced insulin resistance. *Biochimica et Biophysica Acta—Molecular Basis of Disease*.

[37] Kahn S. E., Hull R. L., Utzschneider K. M. (2006). Mechanisms linking obesity to insulin resistance and type 2 diabetes. *Nature*.

[38] Parameswaran N., Patial S. (2010). Tumor necrosis factor-a signaling in macrophages. *Critical Reviews in Eukaryotic Gene Expression*.

[39] Yudkin J. S., Kumari M., Humphries S. E., Mohamed-Ali V. (2000). Inflammation, obesity, stress and coronary heart disease: is interleukin-6 the link?. *Atherosclerosis*.

[40] Wellen K. E., Hotamisligil G. S. (2005). Inflammation, stress, and diabetes. *Journal of Clinical Investigation*.

